# Crystal structure of di­chlorido­{2-[(2-hy­droxyeth­yl)(pyridin-2-ylmeth­yl)amino]­ethano­lato-κ^4^ *N*,*N*′,*O*,*O*′}iron(III) dihydrate from synchrotron data

**DOI:** 10.1107/S1600536814022089

**Published:** 2014-10-11

**Authors:** Jong Won Shin, Dae-Woong Kim, Dohyun Moon

**Affiliations:** a Beamline Department, Pohang Accelerator Laboratory/POSTECH 80, Pohang 790-784, Republic of Korea

**Keywords:** crystal structure, tetra­dentate ligand, Fe^III^ high-spin complex

## Abstract

In the title compound, [Fe(C_10_H_15_N_2_O_2_)Cl_2_]·2H_2_O, the Fe^III^ ion is coordinated by two N and two O atoms of the tetra­dentate 2-{(2-hy­droxy­eth­yl)(pyridin-2-ylmeth­yl)amino}­ethano­late ligand and by two chloride anions, resulting in a distorted octa­hedral coordination sphere. The average Fe—*X* (*X* = ligand N and O atoms) and Fe—Cl bond lengths are 2.10 and 2.32 Å, respectively. In the crystal, duplex O—H⋯O hydrogen bonds between the hydroxyl and eth­oxy groups of two neighbouring complexes give rise to a dimeric unit. The dimers are connected to the lattice water mol­ecules (one of which is equally disordered over two sets of sites) through O—H⋯Cl hydrogen bonds, forming undulating sheets parallel to (010). Weak C—H⋯Cl hydrogen bonds are also observed.

## Abstract 2

The Fe^III^ ion in the title compound shows a slightly distorted FeCl_2_N_2_O_2_ octa­hedral coordination geometry. In the crystal, two complex mol­ecules are linked by duplex O—H⋯O hydrogen bonds. Additional hydrogen-bonding inter­actions lead to the formation of undulating sheets parallel to (010).

## Chemical context   

Tetra­dentate ligands including pyridine and hydroxyl groups have attracted considerable attention in chemistry and mat­erials science (Paz *et al.*, 2012 ▶; Li *et al.*, 2007 ▶). These ligands are able to form multinuclear complexes with various transition metal ions, leading to dimeric, trimeric, tetra­meric or polymeric structures through the deprotonation of hydroxyl groups (Shin *et al.*, 2010 ▶; Han *et al.*, 2009 ▶). Such multinuclear complexes have potential applications in catalysis and magnetic materials. For example, Fe^III^ and Co^II/III^ complexes with amino­ethanol moieties have been studied as oxidation catalysts of various olefins and investigated due to their magnetic properties (Shin *et al.*, 2011 ▶, 2014 ▶). Moreover, Mn^II/III^ complexes containing hydroxyl substituents exhibit excellent single-mol­ecular magnetic properties due to magnetic spin-orbit anisotropy (Wu *et al.*, 2010 ▶).
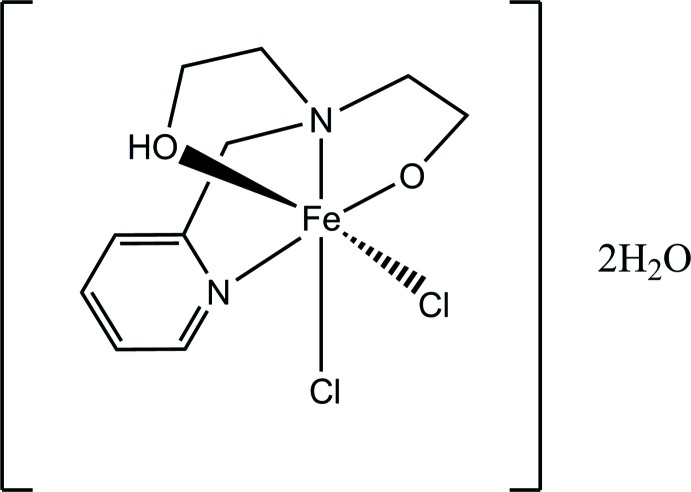


Here, we report the synthesis and crystal structure of a complex with six-coordinate Fe^III^ constructed from the tetra­dentate ligand 2-[(2-hy­droxy­eth­yl)(pyridin-2-ylmeth­yl)amino]­ethanol (H_2_pmide; C_10_H_17_N_2_O_2_) and chloride anions, [Fe(Hpmide)Cl_2_]·2H_2_O, (I).

## Structural commentary   

**Figure 1:**
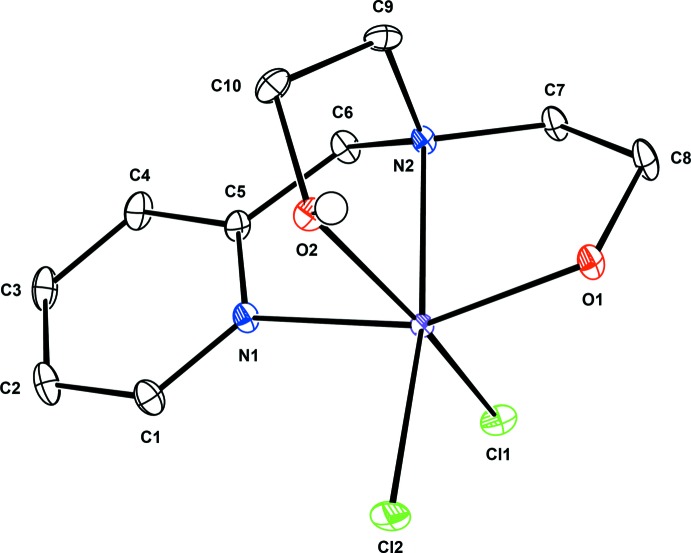
View of the mol­ecular structure of the title compound, showing the atom-labelling scheme, with displacement ellipsoids drawn at the 50% probability level. H atoms and lattice water mol­ecules are omitted for clarity except for the H atom of the hydroxyl group.

A view of the mol­ecular structure of compound (I) is shown in Fig. 1 ▶. The coordination sphere of the Fe^III^ ion can be described as distorted octa­hedral, consisting of the two N atoms and two O atoms from the Hpmide ligand, and two chloride anions. The chloride anions are *trans* to the deprotonated eth­oxy O atom and the N atom of the pyridine group of the Hpmide ligand, respectively, and coordinate in *cis* position to each other. The average Fe—*X*_Hpmide_ (*X* = N, O) bond length is 2.10 Å and the Fe—Cl bond lengths are 2.2773 (5) (equatorial) and 2.3581 (7) (axial) Å. Both the average Fe—N (2.182 Å) and Fe—O (2.010 Å) distances in (I) are comparable to those found in related N_2_O_2_-chelated high-spin Fe^III^ complexes (Shin *et al.*, 2014 ▶; Cappillino *et al.*, 2012 ▶). The bite angles of the five-membered chelate rings in (I) range from 76.59 (5) to 81.45 (4)°.

## Supra­molecular features   

**Figure 2:**
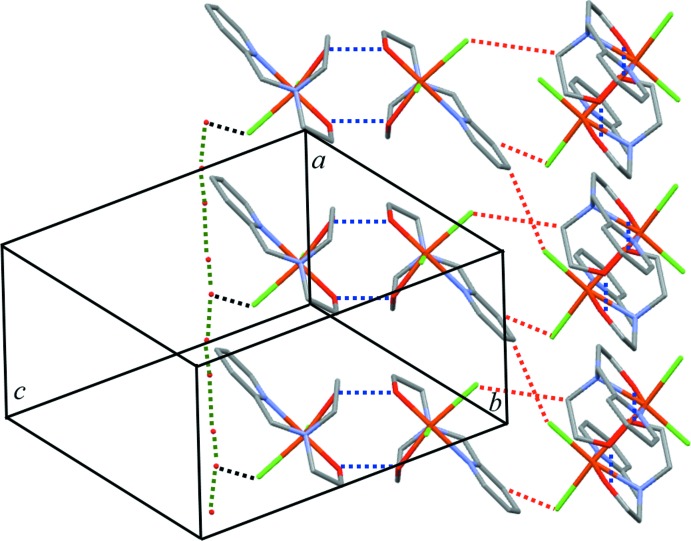
View of the crystal packing of the title compound, with inter­molecular O—H⋯O hydrogen bonds between Fe^III^ complex mol­ecules drawn as blue dashed lines. C—H⋯Cl hydrogen bonds are indicated as red dashed lines; water mol­ecules and chloride anions are also connected through O—H⋯O hydrogen bonds (black dashed lines).

The hydroxyl substituent of the Hpmide ligand forms a strong hydrogen bond with the O atom of the deprotonated eth­oxy group of a neighbouring mol­ecule. These duplex inter­actions lead to a dinuclear dimeric unit. The dimers are linked through O—H⋯Cl inter­actions to the lattice water mol­ecules, that are likewise connected to each other through O—H⋯O hydrogen bonds. All these hydrogen-bonding inter­actions (Steed & Atwood, 2009 ▶) lead to the formation of undulating sheets parallel to (010). Further weak hydrogen bonding between pyridine and methyl H atoms and chloride anions stabilizes this arrangement (Fig. 2 ▶ and Table 1 ▶).

## Database survey   

A search of the Cambridge Structural Database (Version 5.35, November 2013 with three updates; Groom & Allen, 2014 ▶) indicated that five complexes derived from the H_2_pmide ligand have been reported. These include Ni^II^ and Mn^II/III^; Fe^III^ complexes have been studied for their magnetic properties and catalytic effects (Saalfrank *et al.*, 2001 ▶; Wu *et al.*, 2010 ▶; Shin *et al.*, 2014 ▶).

## Synthesis and crystallization   

The H_2_pmide ligand was prepared following a previously reported method (Wu *et al.*, 2010 ▶). Compound (I) was prepared as follows: to a MeOH solution (4 ml) of FeCl_2_·4H_2_O (81 mg, 0.408 mmol) was added dropwise a MeOH solution (3 ml) of H_2_pmide (80 mg, 0.408 mmol). The colour became yellow, and then the solution was stirred for 30 min at room temperature. Yellow crystals of (I) were obtained by diffusion of diethyl ether into the yellow solution for several days, and were collected by filtration and washed with diethyl ether and dried in air. Yield: 67 mg (46%). Elemental analysis calculated for C_10_H_15_Cl_2_FeN_2_O_2_: C 37.30, H 4.70, N 8.70%; found: C 37.19, H 4.58, N 8.78%.

## Refinement   

Crystal data, data collection and structure refinement details are summarized in Table 2 ▶. H atoms attached to C atoms were placed in geometrically idealized positions and constrained to ride on their parent atoms, with C—H distances of 0.95 (aromatic H atoms) and 0.99 Å (open-chain H atoms) and with *U*_iso_(H) values of 1.2*U*_eq_(C) of the parent atoms. One lattice water mol­ecule (O*W*1) was found to be equally disordered over two positions. The H atoms of this disordered water mol­ecule (H1*W*1 and H1*W*2) were located from difference Fourier maps and refined with restraints and a fixed O—H distances of 0.84 Å, with *U*_iso_(H) values of 1.2*U*_eq_(O). Moreover, the second water mol­ecule (O2*W*) was modelled without hydrogen atoms because difference Fourier maps did not suggest suitable H atoms.

## Supplementary Material

Crystal structure: contains datablock(s) I. DOI: 10.1107/S1600536814022089/wm5071sup1.cif

Structure factors: contains datablock(s) I. DOI: 10.1107/S1600536814022089/wm5071Isup2.hkl

CCDC reference: 1027864

Additional supporting information:  crystallographic information; 3D view; checkCIF report

## Figures and Tables

**Table 1:** Hydrogen-bond geometry (, )

*D*H*A*	*D*H	H*A*	*D* *A*	*D*H*A*
O1*W*1H1*W*1Cl1	0.84(1)	2.51(3)	3.279(4)	152(5)
O1*W*2H2*W*2Cl1	0.84(1)	2.91(5)	3.470(4)	125(5)
O2H1O2O1^i^	0.83(2)	1.69(2)	2.5196(14)	177(2)
O1*W*1H2*W*1O2*W* ^ii^	0.84(1)	2.15(4)	2.876(7)	144(7)
O1*W*2H1*W*2O2*W* ^ii^	0.84(1)	2.06(5)	2.647(8)	126(5)
O1*W*2H1*W*2O2*W* ^iii^	0.84(1)	2.06(3)	2.836(8)	153(6)
C4H4Cl1^iv^	0.95	2.76	3.5962(16)	147
C9H9*A*Cl1^v^	0.99	2.78	3.6371(15)	145
C3H3Cl2^vi^	0.95	2.80	3.5721(16)	139

Symmetry codes: (i) 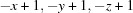; (ii) 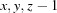; (iii) 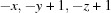; (iv) 
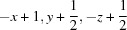
; (v) 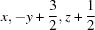; (vi) 
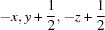
.

**Table 2:** Experimental details

Crystal data	
Chemical formula	[Fe(C_10_H_15_N_2_O_2_)Cl_2_]2H_2_O
*M*_r_	358.02
Crystal system, space group	Monoclinic, *P*2_1_/*c*
Temperature (K)	100
*a*, *b*, *c* ()	7.2690(15), 14.497(3), 14.094(3)
()	95.86(3)
*V* (^3^)	1477.4(5)
*Z*	4
Radiation type	Synchrotron, = 0.62998
(mm^1^)	0.99
Crystal size (mm)	0.10 0.10 0.08

Data collection	
Diffractometer	ADSC Q210 CCD area-detector
Absorption correction	Empirical (using intensity measurements) (*HKL3000sm *SCALEPACK**; Otwinowski Minor, 1997 ▶)
*T*_min_, *T*_max_	0.907, 0.925
No. of measured, independent and observed [*I* > 2(*I*)] reflections	14975, 4056, 3866
*R*_int_	0.021
(sin /)_max_ (^1^)	0.696

Refinement	
*R*[*F* ^2^ > 2(*F* ^2^)], *wR*(*F* ^2^), *S*	0.027, 0.073, 1.04
No. of reflections	4056
No. of parameters	197
No. of restraints	7
H-atom treatment	H atoms treated by a mixture of independent and constrained refinement
_max_, _min_ (e ^3^)	1.51, 0.84

Computer programs: *PAL ADSC Quantum-210 ADX* (Arvai Nielsen, 1983 ▶), *HKL3000sm* (Otwinowski Minor, 1997 ▶), *SHELXS2013/1* and *SHELXL2014/7* (Sheldrick, 2008 ▶), *ORTEP-3 for Windows* and *WinGX* (Farrugia, 2012 ▶).

## supplementary crystallographic information

### Crystal data

**Table:**

[Fe(C_10_H_15_N_2_O_2_)Cl_2_]·2H_2_O	*F*(000) = 740
*M**_r_* = 358.02	*D*_x_ = 1.610 Mg m^−^^3^
Monoclinic, *P*2_1_/*c*	Synchrotron radiation, λ = 0.62998 Å
*a* = 7.2690 (15) Å	Cell parameters from 47717 reflections
*b* = 14.497 (3) Å	θ = 0.4–33.6°
*c* = 14.094 (3) Å	µ = 0.99 mm^−^^1^
β = 95.86 (3)°	*T* = 100 K
*V* = 1477.4 (5) Å^3^	Block, yellow
*Z* = 4	0.10 × 0.10 × 0.08 mm

### Data collection

**Table:**

ADSC Q210 CCD area-detector diffractometer	3866 reflections with *I* > 2σ(*I*)
Radiation source: PLSII 2D bending magnet	*R*_int_ = 0.021
ω scan	θ_max_ = 26.0°, θ_min_ = 2.5°
Absorption correction: empirical (using intensity measurements) (*HKL3000sm *SCALEPACK**; Otwinowski & Minor, 1997)	*h* = −10→10
*T*_min_ = 0.907, *T*_max_ = 0.925	*k* = −19→19
14975 measured reflections	*l* = −19→19
4056 independent reflections	

### Refinement

**Table:**

Refinement on *F*^2^	7 restraints
Least-squares matrix: full	Hydrogen site location: mixed
*R*[*F*^2^ > 2σ(*F*^2^)] = 0.027	H atoms treated by a mixture of independent and constrained refinement
*wR*(*F*^2^) = 0.073	*w* = 1/[σ^2^(*F*_o_^2^) + (0.0354*P*)^2^ + 1.6679*P*] where *P* = (*F*_o_^2^ + 2*F*_c_^2^)/3
*S* = 1.04	(Δ/σ)_max_ = 0.001
4056 reflections	Δρ_max_ = 1.51 e Å^−^^3^
197 parameters	Δρ_min_ = −0.84 e Å^−^^3^

### Special details

**Table:**

Geometry. All e.s.d.'s (except the e.s.d. in the dihedral angle between two l.s. planes) are estimated using the full covariance matrix. The cell e.s.d.'s are taken into account individually in the estimation of e.s.d.'s in distances, angles and torsion angles; correlations between e.s.d.'s in cell parameters are only used when they are defined by crystal symmetry. An approximate (isotropic) treatment of cell e.s.d.'s is used for estimating e.s.d.'s involving l.s. planes.

### Fractional atomic coordinates and isotropic or equivalent isotropic displacement parameters (Å^2^)

**Table:**

	*x*	*y*	*z*	*U*_iso_*/*U*_eq_	Occ. (<1)
Fe1	0.49921 (2)	0.59458 (2)	0.35450 (2)	0.00581 (6)	
Cl1	0.66209 (5)	0.61966 (2)	0.22045 (2)	0.01181 (8)	
Cl2	0.32215 (5)	0.47400 (2)	0.29547 (2)	0.01270 (8)	
O1	0.70234 (13)	0.53542 (7)	0.42787 (7)	0.00933 (18)	
O2	0.33434 (14)	0.60360 (7)	0.46844 (7)	0.00925 (18)	
H1O2	0.322 (3)	0.5590 (12)	0.5041 (13)	0.011*	
N1	0.32550 (15)	0.70945 (8)	0.30279 (8)	0.0085 (2)	
N2	0.62789 (15)	0.71338 (8)	0.43157 (8)	0.0075 (2)	
C1	0.15712 (19)	0.70132 (10)	0.25359 (10)	0.0117 (2)	
H1	0.1099	0.6415	0.2383	0.014*	
C2	0.05082 (19)	0.77769 (11)	0.22469 (10)	0.0143 (3)	
H2	−0.0669	0.7703	0.1896	0.017*	
C3	0.1193 (2)	0.86521 (11)	0.24795 (11)	0.0156 (3)	
H3	0.0492	0.9185	0.2288	0.019*	
C4	0.2913 (2)	0.87365 (10)	0.29950 (11)	0.0130 (3)	
H4	0.3401	0.9328	0.3169	0.016*	
C5	0.39138 (18)	0.79407 (9)	0.32537 (10)	0.0089 (2)	
C6	0.58321 (19)	0.79876 (9)	0.37700 (10)	0.0107 (2)	
H6A	0.6743	0.8079	0.3302	0.013*	
H6B	0.5917	0.8522	0.4210	0.013*	
C7	0.82796 (18)	0.69147 (9)	0.43890 (10)	0.0102 (2)	
H7A	0.8958	0.7310	0.4879	0.012*	
H7B	0.8770	0.7035	0.3771	0.012*	
C8	0.85570 (19)	0.58946 (9)	0.46615 (11)	0.0125 (3)	
H8A	0.9699	0.5663	0.4416	0.015*	
H8B	0.8706	0.5835	0.5365	0.015*	
C9	0.56136 (19)	0.71512 (10)	0.52794 (10)	0.0113 (2)	
H9A	0.5759	0.7781	0.5548	0.014*	
H9B	0.6373	0.6726	0.5708	0.014*	
C10	0.35997 (19)	0.68663 (10)	0.52331 (10)	0.0117 (2)	
H10A	0.3250	0.6762	0.5885	0.014*	
H10B	0.2804	0.7362	0.4932	0.014*	
O1W1	0.4145 (8)	0.5368 (3)	0.0321 (3)	0.0527 (11)	0.5
H1W1	0.444 (8)	0.551 (5)	0.0896 (16)	0.063*	0.5
H2W1	0.300 (3)	0.526 (6)	0.025 (4)	0.063*	0.5
O1W2	0.3007 (7)	0.5629 (4)	0.0504 (3)	0.0549 (11)	0.5
H1W2	0.190 (3)	0.546 (5)	0.041 (4)	0.066*	0.5
H2W2	0.314 (8)	0.593 (4)	0.101 (3)	0.066*	0.5
O2W	0.0911 (9)	0.4335 (4)	0.9619 (4)	0.182 (2)	

### Atomic displacement parameters (Å^2^)

**Table:**

	*U*^11^	*U*^22^	*U*^33^	*U*^12^	*U*^13^	*U*^23^
Fe1	0.00713 (10)	0.00133 (10)	0.00853 (10)	−0.00039 (6)	−0.00140 (6)	0.00067 (6)
Cl1	0.01740 (15)	0.00679 (15)	0.01158 (14)	−0.00204 (11)	0.00321 (11)	0.00141 (10)
Cl2	0.01370 (15)	0.00686 (15)	0.01705 (15)	−0.00509 (10)	−0.00075 (11)	−0.00279 (11)
O1	0.0089 (4)	0.0039 (4)	0.0146 (4)	−0.0005 (3)	−0.0017 (3)	0.0029 (3)
O2	0.0115 (4)	0.0047 (4)	0.0117 (4)	−0.0016 (3)	0.0018 (3)	0.0014 (3)
N1	0.0085 (5)	0.0051 (5)	0.0115 (5)	0.0001 (4)	−0.0005 (4)	0.0023 (4)
N2	0.0082 (5)	0.0031 (5)	0.0105 (5)	−0.0002 (4)	−0.0016 (4)	0.0019 (4)
C1	0.0100 (6)	0.0114 (6)	0.0132 (6)	−0.0008 (5)	−0.0009 (5)	0.0036 (5)
C2	0.0083 (5)	0.0179 (7)	0.0165 (6)	0.0022 (5)	−0.0004 (5)	0.0072 (5)
C3	0.0129 (6)	0.0134 (7)	0.0206 (7)	0.0072 (5)	0.0025 (5)	0.0074 (5)
C4	0.0144 (6)	0.0056 (6)	0.0191 (6)	0.0035 (5)	0.0025 (5)	0.0040 (5)
C5	0.0102 (5)	0.0045 (6)	0.0121 (6)	0.0012 (4)	0.0010 (4)	0.0023 (4)
C6	0.0120 (6)	0.0019 (5)	0.0172 (6)	−0.0010 (4)	−0.0033 (5)	0.0028 (4)
C7	0.0072 (5)	0.0068 (6)	0.0161 (6)	−0.0016 (4)	−0.0022 (4)	0.0025 (4)
C8	0.0079 (5)	0.0077 (6)	0.0207 (7)	−0.0005 (4)	−0.0041 (5)	0.0046 (5)
C9	0.0156 (6)	0.0083 (6)	0.0099 (6)	−0.0024 (5)	0.0001 (5)	−0.0024 (4)
C10	0.0147 (6)	0.0070 (6)	0.0140 (6)	0.0008 (5)	0.0041 (5)	−0.0012 (5)
O1W1	0.095 (4)	0.035 (2)	0.0249 (16)	−0.019 (2)	−0.0105 (19)	0.0025 (14)
O1W2	0.066 (3)	0.069 (3)	0.0270 (18)	−0.008 (2)	−0.0078 (18)	−0.0038 (18)
O2W	0.229 (6)	0.182 (5)	0.138 (4)	−0.037 (5)	0.023 (4)	0.009 (4)

### Geometric parameters (Å, º)

**Table:**

Fe1—O1	1.9165 (11)	C4—C5	1.3925 (18)
Fe1—O2	2.1036 (12)	C4—H4	0.9500
Fe1—N1	2.1704 (12)	C5—C6	1.5071 (19)
Fe1—N2	2.1939 (12)	C6—H6A	0.9900
Fe1—Cl2	2.2773 (5)	C6—H6B	0.9900
Fe1—Cl1	2.3581 (7)	C7—C8	1.5360 (19)
O1—C8	1.4229 (16)	C7—H7A	0.9900
O2—C10	1.4326 (17)	C7—H7B	0.9900
O2—H1O2	0.829 (15)	C8—H8A	0.9900
N1—C5	1.3433 (17)	C8—H8B	0.9900
N1—C1	1.3488 (17)	C9—C10	1.516 (2)
N2—C6	1.4759 (17)	C9—H9A	0.9900
N2—C7	1.4818 (17)	C9—H9B	0.9900
N2—C9	1.4878 (18)	C10—H10A	0.9900
C1—C2	1.387 (2)	C10—H10B	0.9900
C1—H1	0.9500	O1W1—H1W1	0.842 (10)
C2—C3	1.390 (2)	O1W1—H2W1	0.842 (10)
C2—H2	0.9500	O1W2—H1W2	0.842 (10)
C3—C4	1.386 (2)	O1W2—H2W2	0.839 (10)
C3—H3	0.9500		
			
O1—Fe1—O2	94.79 (4)	C3—C4—H4	120.6
O1—Fe1—N1	156.10 (4)	C5—C4—H4	120.6
O2—Fe1—N1	81.45 (4)	N1—C5—C4	122.05 (13)
O1—Fe1—N2	79.52 (5)	N1—C5—C6	116.46 (11)
O2—Fe1—N2	79.66 (4)	C4—C5—C6	121.45 (12)
N1—Fe1—N2	76.59 (5)	N2—C6—C5	110.93 (10)
O1—Fe1—Cl2	103.25 (4)	N2—C6—H6A	109.5
O2—Fe1—Cl2	88.96 (3)	C5—C6—H6A	109.5
N1—Fe1—Cl2	100.28 (4)	N2—C6—H6B	109.5
N2—Fe1—Cl2	168.51 (3)	C5—C6—H6B	109.5
O1—Fe1—Cl1	94.54 (4)	H6A—C6—H6B	108.0
O2—Fe1—Cl1	166.87 (3)	N2—C7—C8	109.06 (11)
N1—Fe1—Cl1	86.28 (3)	N2—C7—H7A	109.9
N2—Fe1—Cl1	92.98 (3)	C8—C7—H7A	109.9
Cl2—Fe1—Cl1	97.869 (19)	N2—C7—H7B	109.9
C8—O1—Fe1	119.26 (8)	C8—C7—H7B	109.9
C10—O2—Fe1	114.30 (8)	H7A—C7—H7B	108.3
C10—O2—H1O2	110.1 (14)	O1—C8—C7	110.95 (11)
Fe1—O2—H1O2	121.2 (14)	O1—C8—H8A	109.4
C5—N1—C1	118.97 (12)	C7—C8—H8A	109.4
C5—N1—Fe1	116.11 (9)	O1—C8—H8B	109.4
C1—N1—Fe1	124.88 (9)	C7—C8—H8B	109.4
C6—N2—C7	112.22 (11)	H8A—C8—H8B	108.0
C6—N2—C9	112.75 (11)	N2—C9—C10	111.01 (11)
C7—N2—C9	110.38 (11)	N2—C9—H9A	109.4
C6—N2—Fe1	109.90 (8)	C10—C9—H9A	109.4
C7—N2—Fe1	103.42 (8)	N2—C9—H9B	109.4
C9—N2—Fe1	107.66 (8)	C10—C9—H9B	109.4
N1—C1—C2	122.02 (13)	H9A—C9—H9B	108.0
N1—C1—H1	119.0	O2—C10—C9	108.90 (11)
C2—C1—H1	119.0	O2—C10—H10A	109.9
C1—C2—C3	118.93 (13)	C9—C10—H10A	109.9
C1—C2—H2	120.5	O2—C10—H10B	109.9
C3—C2—H2	120.5	C9—C10—H10B	109.9
C4—C3—C2	119.12 (13)	H10A—C10—H10B	108.3
C4—C3—H3	120.4	H1W1—O1W1—H2W1	108 (3)
C2—C3—H3	120.4	H1W2—O1W2—H2W2	109 (3)
C3—C4—C5	118.90 (14)		
			
C5—N1—C1—C2	0.7 (2)	Fe1—N2—C6—C5	34.62 (13)
Fe1—N1—C1—C2	178.26 (10)	N1—C5—C6—N2	−26.63 (17)
N1—C1—C2—C3	−0.6 (2)	C4—C5—C6—N2	155.71 (13)
C1—C2—C3—C4	−0.2 (2)	C6—N2—C7—C8	−161.67 (11)
C2—C3—C4—C5	0.8 (2)	C9—N2—C7—C8	71.64 (14)
C1—N1—C5—C4	−0.1 (2)	Fe1—N2—C7—C8	−43.28 (12)
Fe1—N1—C5—C4	−177.80 (10)	Fe1—O1—C8—C7	−0.47 (15)
C1—N1—C5—C6	−177.70 (12)	N2—C7—C8—O1	31.76 (16)
Fe1—N1—C5—C6	4.56 (16)	C6—N2—C9—C10	83.81 (14)
C3—C4—C5—N1	−0.7 (2)	C7—N2—C9—C10	−149.79 (11)
C3—C4—C5—C6	176.80 (13)	Fe1—N2—C9—C10	−37.58 (12)
C7—N2—C6—C5	149.11 (12)	Fe1—O2—C10—C9	−35.00 (13)
C9—N2—C6—C5	−85.49 (14)	N2—C9—C10—O2	48.37 (15)

### Hydrogen-bond geometry (Å, º)

**Table:**

*D*—H···*A*	*D*—H	H···*A*	*D*···*A*	*D*—H···*A*
O1*W*1—H1*W*1···Cl1	0.84 (1)	2.51 (3)	3.279 (4)	152 (5)
O1*W*2—H2*W*2···Cl1	0.84 (1)	2.91 (5)	3.470 (4)	125 (5)
O2—H1*O*2···O1^i^	0.83 (2)	1.69 (2)	2.5196 (14)	177 (2)
O1*W*1—H2*W*1···O2*W*^ii^	0.84 (1)	2.15 (4)	2.876 (7)	144 (7)
O1*W*2—H1*W*2···O2*W*^ii^	0.84 (1)	2.06 (5)	2.647 (8)	126 (5)
O1*W*2—H1*W*2···O2*W*^iii^	0.84 (1)	2.06 (3)	2.836 (8)	153 (6)
C4—H4···Cl1^iv^	0.95	2.76	3.5962 (16)	147
C9—H9*A*···Cl1^v^	0.99	2.78	3.6371 (15)	145
C3—H3···Cl2^vi^	0.95	2.80	3.5721 (16)	139

Symmetry codes: (i) −*x*+1, −*y*+1, −*z*+1; (ii) *x*, *y*, *z*−1; (iii) −*x*, −*y*+1, −*z*+1; (iv) −*x*+1, *y*+1/2, −*z*+1/2; (v) *x*, −*y*+3/2, *z*+1/2; (vi) −*x*, *y*+1/2, −*z*+1/2.
